# Adverse short-term effects of ozone on cardiovascular mortalities modified by season and temperature: a time-series study

**DOI:** 10.3389/fpubh.2023.1182337

**Published:** 2023-06-09

**Authors:** Panjun Gao, Yongsheng Wu, Lihuan He, Li Wang, Yingbin Fu, Jinrong Chen, Fengying Zhang, Thomas Krafft, Pim Martens

**Affiliations:** ^1^Department of Health, Ethics and Society, Care and Public Health Research Institute (CAPHRI), Faculty of Health, Medicine and Life Sciences, Maastricht University, Maastricht, Netherlands; ^2^Shenzhen Center for Disease Control and Prevention, Shenzhen, China; ^3^China National Environmental Monitoring Centre, Beijing, China; ^4^Key Laboratory of Land Surface Pattern and Simulation, Institute of Geographical Sciences and Natural Resources Research, Chinese Academy of Sciences, Beijing, China; ^5^University College Venlo, Maastricht University, Venlo, Netherlands

**Keywords:** hypertension, ischemic heart disease, stroke, air pollution, climate change

## Abstract

**Introduction:**

Ambient ozone pollution becomes critical in China. Conclusions on the short-term effects of ozone on cardiovascular mortality have been controversial and limited on cause-specific cardiovascular mortalities and their interactions with season and temperature. This research aimed to investigate the short-term effects of ozone and the modifications of season and temperature on cardiovascular mortality.

**Methods:**

Cardiovascular death records, air pollutants, and meteorological factors in Shenzhen from 2013 to 2019 were analyzed. Daily 1-h maximum of ozone and daily maximum 8-h moving average of ozone were studied. Generalized additive models (GAMs) were applied to evaluate their associations with cardiovascular mortalities in sex and age groups. Effect modifications were assessed by stratifying season and temperature.

**Results:**

Distributed lag impacts of ozone on total cardiovascular deaths and cumulative effects on mortality due to ischemic heart disease (IHD) were most significant. Population under 65 years old was most susceptible. Majority of significant effects were found in warm season, at high temperature, and at extreme heat. Ozone-associated risks in total deaths caused by hypertensive diseases reduced in warm season, while risks in IHD in males increased at high temperature. Extreme heat enhanced ozone effects on deaths caused by CVDs and IHD in the population under 65 years old.

**Discussion:**

The revealed cardiovascular impacts of ozone below current national standard of air quality suggested improved standards and interventions in China. Higher temperature, particularly extreme heat, rather than warm season, could significantly enhance the adverse effects of ozone on cardiovascular mortality in population under 65 years old.

## 1. Introduction

Ozone is a secondary air pollutant, produced from volatile organic compounds (VOCs), and nitrogen oxides (NO_X_) through a series of photochemical reactions under solar radiation. As one of the main air pollutants, it has been identified as a global health hazard (1). Ozone was suggested to increase the short-term risks of the worldwide total mortality (2), and attribute to about 63 million of mortality around the world (3).

Cardiovascular diseases (CVDs), as the death cause ranking first globally (World Health Organization, WHO), have been also reported to be associated with the concentration of ozone. Increasing ozone was suggested to be associated with the admissions (4, 5) and the mortality (6, 7) of overall cardiovascular diseases. Regarding specific cardiovascular diseases, the relative findings were more diverse. Several studies demonstrated the positive associations between ozone and ischemic heart diseases (IHD) (8, 9), while several others did not notice such relationships (4, 10, 11). Similar situations were also found in research related to other cardiovascular diseases, such as myocardial infarction (12–14) and stroke (15, 16).

Ambient ozone pollution has drawn increasing attention in China. The level of ozone in China has been found to increase (17) and China was indicated to be at risks of more severe and frequent high-ozone events compared to other parts of the world (18). The seasonality of ozone and its close associations with meteorological factors were reported to be possibly independent from precursor emissions (19). Thus, the possible influence of climate on ground-level ozone and its relationship with health have been noted considering the aggravation of climate change and extreme weather events. Climate change has been suggested to be associated with the level of ozone and increase the ozone-related risk of total mortality (20). Several national studies in China showed the positive associations of ozone with year of life lost (21) and mortality (16, 22) due to CVDs, but suggested that season and temperature modified differently across China. Extreme weather events have been indicated to increase the level of ozone and such trend will continue in the future (23). Some studies on China have also noticed that the concentration of ozone increased along with heat wave (24, 25). However, despite the suggested increasing influence of extreme temperature events in China, there have been insufficient investigations on their consequences, especially the health effects (26).

In general, the health effects of ozone are demanding more attention because of its high concentration and the larger influence of climate change in the future (20). Nevertheless, current evidence on the short-term effects of ozone specifically on cardiovascular mortality has been controversial and limited on mortalities due to specific cardiovascular causes. Furthermore, it is still less known about the interactions of season and temperature on the associations between ozone and CVD-caused mortalities. In despite of the inconsistent evidence and the instability of ozone, evaluations on the independent effects of ozone are necessary for risk assessment and effective regulations. Therefore, this research aimed to investigate the short-term independent effects of ozone on overall and cause-specific cardiovascular mortalities, and to explore the modifications and differences of season, temperature, and extreme heat on the associations between ozone and cardiovascular mortalities.

## 2. Methods

### 2.1. Study area

This research was conducted in Shenzhen. Shenzhen is a coastal city located in Pearl River Delta (PRD) in southern China. It is one of the cities which first implemented the current national standard of air quality in China (GB3095-2012) since 2013. In addition to daily 1-h maximum of ozone (O_3_-1h), as one of the major modifications, daily maximum 8-h moving average of ozone (O_3_-8h) is introduced in this standard. The real-time hourly concentrations of air pollutants in Shenzhen have been monitored since January 1st, 2013. The datasets of air pollutants could be obtained from China National Environmental Monitoring Centre (CNEMC), which is the institution responsible for nationwide data collection and environment quality evaluation.

The mortality data in Shenzhen could be derived from the death registry system of Shenzhen Centre for Disease Prevention and Control (CDC). The system, as the local part of the national mortality surveillance system, has been developed to register each death occurring in its area of administration. Shenzhen CDC is responsible for the gathering and verification of the deaths registered by healthcare facilities. The death records with permanent addresses in Shenzhen were selected as our research subjects. Therefore, the abundant and high-quality datasets ensure the possibilities of analyses in a comprehensive manner to investigate the independent health effects of ozone.

The weather in Shenzhen is warm and humid because of its subtropical monsoon climate. With a better air quality than most cities in China, Shenzhen has been rewarded as a national model city of environmental protection for its massive efforts and achievements. However, previous research identified that PRD was at the highest risk of severe ozone pollution in China (27). As the most crowded city in PRD and in China, studies on the effects of air pollution on population health in Shenzhen would have great significance.

### 2.2. Environmental variables

The daily concentrations of air pollutants in Shenzhen from 2013 to 2019 were obtained from Shenzhen Environmental Monitoring Centre and CNEMC. The monitoring stations were state-controlled and set according to the technical guidelines developed by the Chinese government. The respective locations of the 11 monitoring sites providing data for this study were sufficiently far away from any traffic intersections, major sources of industrial pollution or any other emission sources. Therefore, the derived data represent the air quality of Shenzhen.

The daily levels of air pollutants were calculated based on the average hourly data across the monitoring sites, including daily 1-h maximum of ozone (O_3_-1h), daily maximum 8-h moving average of ozone (O_3_-8h), nitric oxide (NO), nitrogen dioxide (NO_2_), sulfur dioxide (SO_2_), carbon monoxide (CO), particulate matter with an aerodynamic diameter less than 10 μm (PM_10_). Data from a site would be included only if 75% or more of hourly concentrations of air pollutants for the respective day were available. The dataset from any monitoring site with over 25% data missing during the entire study period would be excluded. There was no such monitoring site in Shenzhen from 2013 to 2019.

The daily levels of meteorological factors were collected from Meteorological Bureau of Shenzhen Municipality, including temperature (Tem), relative humidity (Hum), precipitation (Pre), barometric pressure (BP), wind speed (WS), and sunshine duration (SSD). The monitoring procedure followed the international standards developed by the World Meteorological Organization (WMO).

### 2.3. Mortality data

Death records in Shenzhen from 2013 to 2019 were analyzed based on the comprehensive death registry maintained by Shenzhen CDC, including death date, sex, age, primary cause of death and the corresponding codes of *International Classification of Diseases*, *10th version* (*ICD-10*). The data were divided to investigate the effects of ozone on deaths caused by CVDs (*ICD-10* codes, I00-I99), hypertensive diseases (HBP) (*ICD-10* codes, I10-I15), IHD (*ICD-10* codes, I20-I25), and stroke (STR) (*ICD-10* codes, I60-I69).

### 2.4. Statistical analyses

The statistical analyses were performed to assess the associations between the number of daily deaths and the daily concentrations of ozone. To evaluate the independent effects of ozone, all the meteorological factors and other air pollutants were involved as confounders.

Missing values of the collected environmental factors were imputed using the method of Kalman Smoothing on Structural Time Series “imputeTS” package in R 4.0.2. There was 1 day missing each for O_3_-1h, O_3_-8h, NO_2_, SO_2_, CO, and PM_10_. Data on NO were missing for 95 days. There were no missing values of temperature, relative humidity, or barometric pressure. Data of 1 day each for precipitation, and sunshine duration as well as 214 days for wind speed missed. The relationships between each two environmental factors were analyzed using Spearman correlation.

The mortality records were stratified by sex, age, season, and temperature. Young group included deaths under 65 years old, and senior group included the rest. Season was generally divided by comparing monthly mean temperature with annual mean temperature. Warm season with monthly mean temperature higher than annual mean temperature included months from May to October, and cold season included the rest of months from November to April. Days with unstratified temperature were represented as “ns (not stratified).” The subgroup of days at temperature higher than the median value was described as “high temperature,” otherwise as “low temperature.” Days at temperature not lower than 97.5 percentile were defined as “extreme heat.”

The risk of cardiovascular mortalities related to ambient ozone was analyzed using generalized additive models (GAMs) with penalized splines. As daily mortality followed a Poisson distribution, GAMs with log link and Poisson error were applied. To perform this analysis, the best basic model for each death cause without ozone was firstly built up, and then the main model was developed.

In the basic model, the smoothed spline functions of death date, meteorological variables, and other air pollutants except ozone were involved. The day of week (DOW) was included as a dummy variable in the basic model.

The association between ozone and the logarithmic cardiovascular mortality, following previous research (28), was assumed to be linear. Ozone was introduced into the basic model to analyze the relationship with mortality caused by CVDs. The fitness of the model was evaluated using Akaike’s Information Criterion (AIC). The smaller AIC was, the more the model was preferred. We applied the following GAMs to estimate the log-relative rate β:


$$\log\left[E\left(\mathrm{Yt}\right)\right]=\alpha +\overset{q}{\underset{i=1}{\sum }}\beta i\left(Xi\right)+\overset{p}{\underset{j=1}{\sum }}fj\left(Zj,df\right)+Wt\left(DOW\right)$$

In this equation, E(Yt) refers to the expected death number at day t; β refers to the log-relative rate of mortality associated with a unit increase of ozone; Xi refers to the concentration of ozone at day t; $\overset{p}{\underset{j=1}{\sum }}fj\left(Zj,df\right)$ is the non-parametric spline function of death date, meteorological variables, and other air pollutants; Wt(DOW) is the dummy variable for the day of the week. The degree of freedom (df) was initialized as 9 df/year for death date, and 3 df for all the other variables.

3-day lag effects of ozone on cardiovascular mortality were analyzed. To build the model of lag effects, ozone with different lag structures of single-day lag (distributed lag; L0 to L3, L0 means the current-day concentration and L1 means the concentration on the previous day) and multi-day lag (moving average lag; L01 to L03, L03 means the four-day moving average concentration of the current day and previous 3 days). The current-day levels of meteorological variables and other air pollutants were included in the lag models.

The modifications of season and temperature on the associations between ozone and cardiovascular mortality were analyzed using GAMs with stratification parameters. Current-day effects and lag effects of ozone were examined. The following models were performed to assess the modifications of season and temperature, respectively:


$$\begin{array}{l} \log\left[E\left(\mathrm{Yt}\right)\right]=\alpha +\beta_{1}\left(X\right)+\beta_{2}\left(M_{k}\right)+\beta_{3}\left(X:M_{k}\right)\\ \;\;\;\;\;\;\;\;\;\;\;\;\;\;\;\;\;\;\;\;\;\;\;\;\;\;\;\;\;+\overset{p}{\underset{j=1}{\sum }}fj\left(Zj,df\right)+Wt\left(DOW\right) \end{array}$$

$\beta_{1}$ corresponds to the effects of ozone; X corresponds to the concentrations of ozone;$\;\beta_{2}$ corresponds to the effects of season, temperature, or extreme heat; $M_{k}$ is the current level of modification term (season, temperature, or extreme heat); $\beta_{3}$ corresponds to the interactive effects of ozone and season or temperature. $\overset{p}{\underset{j=1}{\sum }}fj\left(Zj,df\right)$ is the non-parametric spline function of other variables including death date, meteorological variables, and other air pollutants. Wt(DOW) is the dummy variable for the day of the week. The effects of ozone on days in warm season, with high temperature, or with extreme heat were derived from $\beta_{1}$ + $\beta_{3}$. The confidence intervals were obtained using the method for interaction terms (29).

The “mgcv” package in R 4.0.2 (R Foundation for Statistical Computing, Vienna, Austria) was used to perform all the analyses. Relative risk (RR) of death number per 10 μg/m^3^ increase of ozone (RR=$e^{\beta \times \Delta c}$, where Δc is 10 μg/m^3^ of ozone) was used to indicate the effects of ozone on cardiovascular mortality. A *p* value less than 0.05 was considered to be statistically significant.

## 3. Results

### 3.1. Descriptive statistics

The general description of ozone, meteorological factors, and cardiovascular mortalities in total population has been shown in Tables 1, 2, and more relative details can be found in Supplementary Tables S1, S2.

**Table 1:** Annual rate of overall cardiovascular mortality in Shenzhen (per 10,000 person).

Year	2013	2014	2015	2016	2017	2018	2019
Overall CVD deaths	1,684	2,203	1,846	4,527	4,761	5,079	5,104
Annual rate	1.6	2.0	1.6	3.8	3.8	3.9	3.8

**Table 2:** General information of environmental factors and total cardiovascular mortalities.

	Mean	SD	Minimum	25%	Median	75%	Maximum
O_3_-1h (μg/m^3^)	98.4	42.8	18.0	65.0	91.0	123.0	290.0
O_3_-8h (μg/m^3^)	81.1	36.3	14.0	52.0	75.0	103.0	246.0
Tem (°C)	23.5	5.4	3.5	19.5	24.8	28.1	33.0
Hum (%)	75.6	13.1	19.0	69.5	78.0	84.5	100.0
Pre (mm)	5.3	15.9	0.0	0.0	0.0	1.3	187.8
BP (hPa)	1005.5	6.5	983.1	1000.7	1005.5	1010.4	1027.2
WS (m/s)	2.0	0.7	0.4	1.4	1.8	2.3	6.1
SSD (h)	5.2	3.8	0.0	1.4	5.5	8.7	12.5
CVD	9.9	5.4	0	5	9	14	33
HBP	0.9	1.0	0	0	1	1	6
IHD	4.0	3.0	0	2	3	6	17
STR	3.6	2.4	0	2	3	5	14

The daily concentrations of O_3_-1h and O_3_-8h were 98.4 and 81.1 μg/m^3^. The median value of temperature was 24.8°C, and was used as the cut-off point for temperature stratification. The 97.5 percentile of temperature was 30.4°C, not lower than which was defined as extreme heat. During the study period, there were 67 days in total at extreme heat. Regardless of different indicators, the levels of ozone in warm season and at high temperature were higher than those in cold season and at low temperature, respectively. During the days at extreme heat, ozone reached its highest average level.

**Figure 1:**
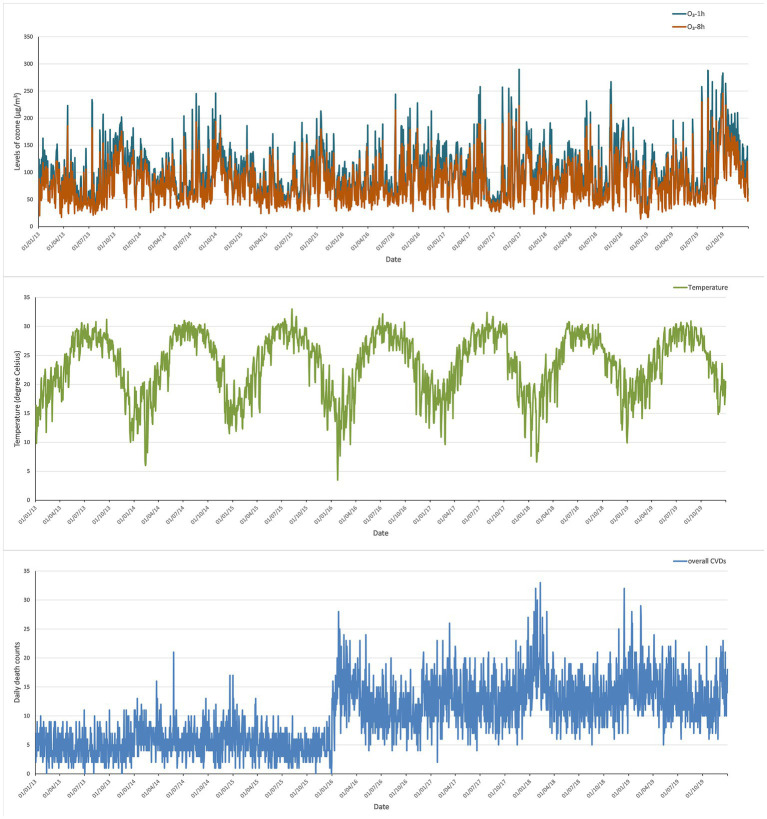
Temporal changes of ozone, temperature, and daily deaths due to overall CVDs in Shenzhen from 2013 to 2019.

The temporal changes of two ozone indicators, O_3_-1h and O_3_-8h, temperature, and the daily deaths caused by overall CVDs were displayed in Figure 1. Overall, the concentration of ozone showed increasing trend from 2013 to 2019. The lowest level of ozone generally happened in June, while the highest level of ozone occurred in Septembers of 2013 and 2019.

The numbers of overall and cause-specific cardiovascular mortalities in different sex and age groups are shown in Supplementary Table S2. Despite of sex and age, IHD and stroke were the major causes of cardiovascular deaths. In general, there were no significant differences in the daily death counts between cold season and low temperature, between warm season and high temperature.

**Figure 2:**
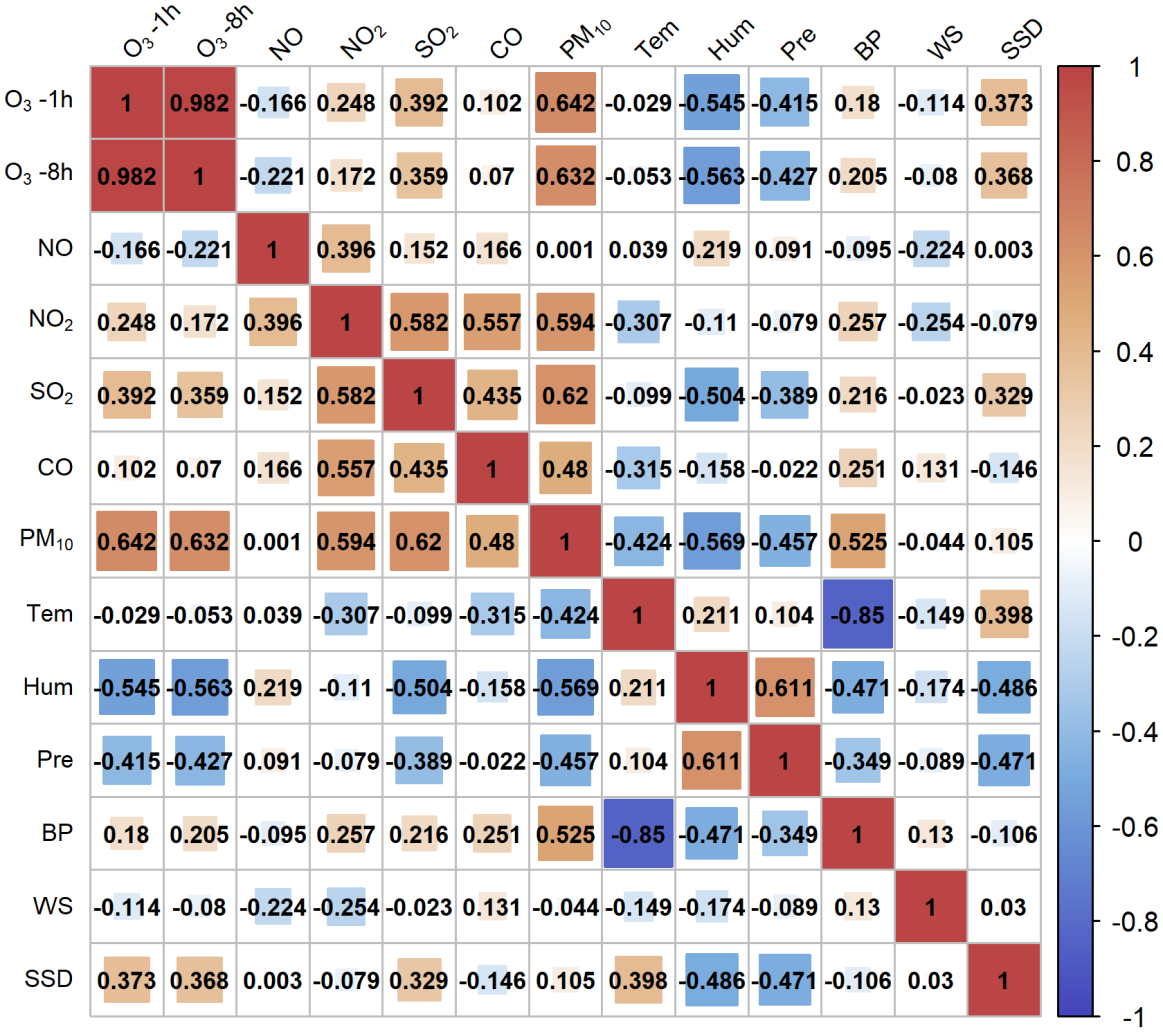
Spearman correlation between air pollutants and meteorological factors.

Figure 2 shows the Spearman correlations between ozone and meteorological factors. Increasing ozone was significantly associated with higher barometric pressure and longer sunshine duration. Temperature, relative humidity, precipitation, and wind speed were negatively related to the concentration of ozone.

### 3.2. Effects of ozone and modifications of season and temperature on cardiovascular mortality

**Figure 3:**
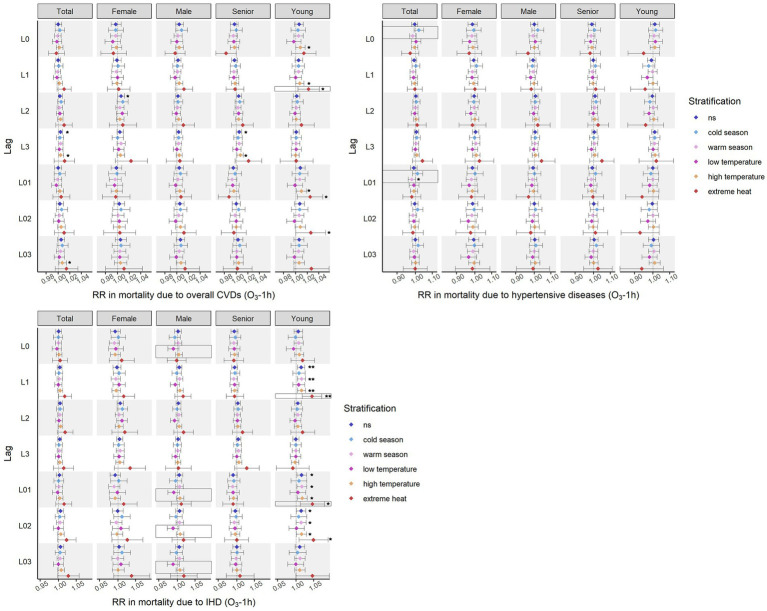
Effects on cardiovascular mortality identified using O_3_-1h as indicator. *p < 0.05; **p < 0.01; in box refers to the interaction term with statistical significance (warm season vs. cold season, low temperature vs. high temperature, extreme heat vs. not extreme heat, *p* value <0.05).

**Figure 4:**
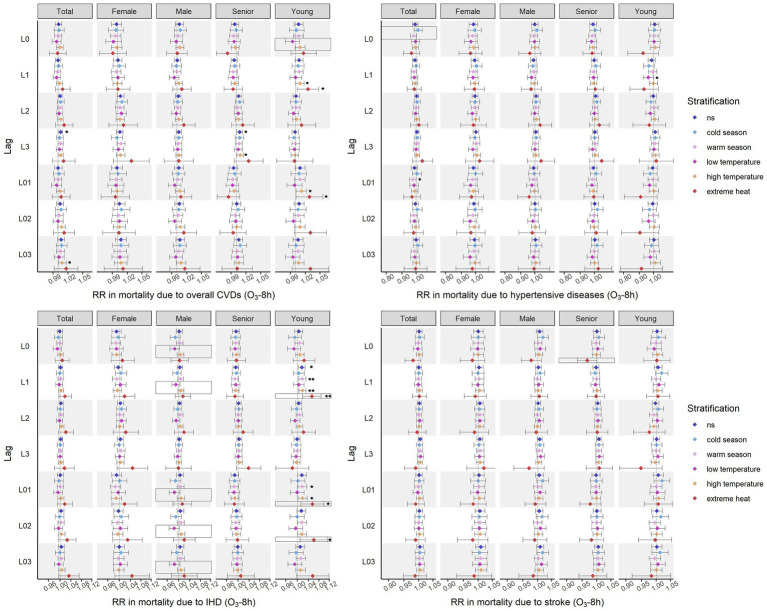
Effects on cardiovascular mortality identified using O_3_-8h as indicator. *p < 0.05; **p < 0.01; in box refers to the interaction term with statistical significance (warm season vs. cold season, low temperature vs. high temperature, extreme heat vs. not extreme heat, *p* value <0.05).

Figures 3, 4 shows the effects of ozone on cardiovascular mortality and the modifications of season and temperature. Slightly more effects were revealed when using O_3_-1h as indicator than those using O_3_-8h. In general, the impacts of ozone on total cardiovascular deaths and mortality due to IHD were most obvious, and population under 65 years old was most susceptible. Majority of the significant effects were found in warm season, days with high temperature, and days with extreme heat. The lag windows during which ozone significantly affected each cardiovascular cause of death showed inconsistency.

Significant effect modifications on the associations between ozone and cardiovascular mortality, were observed in both seasons and temperature strata, yet the modification of temperature was more obvious. Additionally, the detected relationships during each lag window were unstable in the stratifications of temperature and season. Though the days of extreme heat were part of the days with high temperature, not all the positive associations noticed in days at high temperature were also revealed in extreme heat. Similarly, though one of the main meteorological features in warm season was higher temperature, not all the significant effects reported in days at higher temperature were also uncovered in warm season.

#### 3.2.1. Effects identified using O_3_-1h as indicator

The identified effects of ozone on mortality due to CVDs when using O_3_-1h as indicator are presented as Figure 3. Since there were no significant effects found between O_3_-1h and deaths caused by stroke, relevant information is not known in Figure 3.

The level of O_3_-1h was positively related to mortality due to overall CVDs in each lag window. The distributed lag effects of O_3_-1h on total cardiovascular mortality were more obvious than the cumulative effects, with larger number of significant associations and influencing more population groups. Most of the noted associations were identified in days at higher temperature. Extreme heat significantly increased the risk of CVD-caused mortality related to O_3_-1h with 1-day lag. Interestingly, the higher risks of CVD-caused deaths identified using O_3_-1h of L0, L3, and L03 at high temperature were not observed in days with extreme heat. The associations between O_3_-1h at L1 and CVD-caused mortality in the young were insignificant in warm season, but significant at high temperature and extreme heat.

Regarding the specific cardiovascular causes of mortality, the significant effects of O_3_-1h were identified on mortality due to hypertensive diseases in the total population and IHD only in the population under 65 years old. Only was a significantly cumulative and negative association found between O_3_-1h of L01 and deaths caused by hypertensive diseases in the total population. Season was a modifier on L0 and L01. The majority of significant impacts on cause-specific cardiovascular mortality was found in IHD-caused mortality in the young population. The cumulative influence of O_3_-1h on IHD-caused deaths was more obvious than the distributed influence. Extreme heat increased the risks of IHD mortality related to O_3_-1h of L1 and L01. Although without significant impacts revealed, the modification of temperature on O_3_-1h-related IHD mortality were shown in males on the current day, L01, L02, and L03.

#### 3.2.2. Effects identified using O_3_-8h as indicator

The effects of ozone on cardiovascular mortality using O_3_-8h as indicator are shown as Figure 4.

The impacts of O_3_-8h on overall cardiovascular deaths was observed in each lag windows except for L0, L2, and L02. The most significant associations were reported between O_3_-8h of L3 and CVD-caused mortality. Unstable relationships were shown at higher temperature. Statistically positive associations at L3 in the senior population and at L03 in the total population were noted at high temperature but not at extreme heat. The modification effect was only found to be significant between temperature groups on the CVD-caused deaths of the young population with the level of O_3_-8h on the current day.

Significant effects of O_3_-8h were uncovered on mortality due to each specific cardiovascular cause, except stroke. O_3_-8h was negatively related to mortality due to hypertensive diseases in the population under 65 years old at low temperature with 1 day lagged and in the total population in warm season with one-day moving-average lagged. Season was identified as a modifier of O_3_-8h-related deaths caused by HBP hypertensive diseases in the total population on the current day. The effects of O_3_-8h on IHD-caused mortality were also found only in the young. The impacts at L02 were observed at high temperature and extreme heat but not in warm season. The modification effects of high temperature and extreme heat were considerable for deaths caused by IHD in the male and in the young deaths, respectively. Without significant relationships discovered between stroke mortality and O_3_-8h, extreme heat still showed a protective impact on their potential associations.

## 4. Discussion

The average levels of ozone with two indicators, O_3_-1h and O_3_-8h, in Shenzhen from 2013 to 2019 were under the national standards for city, 200 and 160 μg/m^3^, respectively (GB3095-2012). However, in addition to the independent health impacts of ozone with current level (O_3_-8h, 81.1 μg/m^3^), the enhanced effects of ozone under extreme heat were still noted in our research. The Air Quality guideline proposed by WHO (30) in 2021 is below the current Chinese national standard with 60 μg/m^3^ for O_3_-8h, including two interims of 100 and 70 μg/m^3^ in the peak season. Our findings confirmed the WHO recommendation, supporting the need for further adjustments of the national air quality standard and prompting more interventions reducing ozone pollution in China.

The meteorological factors which influenced the concentration of ozone most in our study were relative humidity, precipitation, and sunshine duration. In our research, ozone increased along with lower humidity, lower precipitation, and longer sunshine duration, and the relationship between temperature and ozone was negative and limited. However, a nationwide study in China suggested temperature, humidity, and sunshine duration to influence ozone most (19). Another investigation in Spain determined humidity, wind speed, and temperature as the most influential factors of ozone (31). The regional difference may be related to the specific climate conditions in different areas, such as the weather clusters and the weather frequency change (32).

In our research, overall cardiovascular deaths and mortality due to IHD were affected by ozone the most, while the effects of ozone on mortality due to hypertensive diseases and stroke were relatively insignificant. However, findings from other studies were not all consistent with ours. Though many previous investigations in population observed the significant effects of ozone on cardiovascular health, with a maximum daily 8-h ozone of 93.5 μg/m^3^, one study in Prague did not underline such relationships between ozone and either mortality or hospital admissions due to CVDs (33). Research in Hefei, China, with O_3_-8h of 79.08 μg/m^3^, did not uncover any significant effects of ozone on cardiovascular mortality, either (34). Relative results of mortality due to specific cardiovascular cause were more diverse. There was research reporting significant effects of ozone on deaths caused by hypertensive diseases (16), IHD (16), and stroke (16, 35), while other did not find such relationships of ozone with hypertension (15), IHD (10) or stroke (10, 15). Additionally, previous research underlined significant impacts of ozone on the older adults (14, 36, 37). But our research raises the attention to the population under 65 years old regarding ozone-related cardiovascular events.

More positive associations between ozone and cardiovascular mortality were noticed in warmer season and at higher temperature. Nevertheless, after the statistical tests, more significant effects modifications were verified in the stratifications of temperature. Therefore, regarding the short-term effects of ozone, temperature could be a more sensitive modifier than season.

Specifically in terms of season, most of significant effects of ozone were identified in warm season in our research. These findings were in consistency with several other studies. Though unstable, positive relationships between ozone and cardiovascular mortality were reported in the entire year and in warm season in Beijing, China (38). Similar results were also demonstrated in France (10) and in another study covering Europe (39). However, modification effects of season on ozone-related HBP deaths identified in our research turned to show a protective impact in warm season. These results were in line with a study which was conducted only in warm season observed negative relationships between ozone and cardiovascular deaths in population above 64 years old (11). Nevertheless, there were other investigations revealing different associations between ozone and CVD-caused deaths in different seasons. Rather than warm season, studies in Shanghai (35) and Zhengzhou, China (40) found the significant associations between ozone and cardiovascular mortality in the cold season. Moreover, a study in Iran did not observe the difference of ozone-related cardiovascular deaths between warm and cold season (41).

Regarding the modification of temperature, we discovered more significant impacts and greater risks of ozone in days with high temperature and days with extreme heat. The highest relative risks were revealed at extreme heat when applicable. There were several studies in line with our results. Enhanced cardiovascular effects of ozone at higher temperature (>75 percentile) were also observed in a nationwide study in China (42), an European study covering eight urban areas (43), and a study covering nine French cities (10). Different from the findings of these studies, in the nationwide research of the US, both low and high temperature (1st and 2nd tertiles as cut-offs) could raise the cardiovascular risks related to ozone (44). Regarding the modification of heat on the association between ozone and cardiovascular mortality, relative evidence was more limited and inconsistent. In our research, the increased risks of CVD-caused deaths at extreme heat (not less than 97.5 percentile) were only noticed in the population under 65 years old. With the same cut-off defining heat, the aforementioned French study did not report any significant effects of ozone on cardiovascular mortality at heat (10).

The differences among relative investigations could be explained by several reasons. First, the reported concentrations of ozone varied spatially and temporally. Second, the study designs may have some influence on the results, such as the categorisations of season and temperature, research in a particular group of people. Third, different research considered various confounders. Some studies involved the socioeconomic status of the study population. Temperature and relative humidity were mostly involved, while we also counted in precipitation, barometric pressure, wind speed and sunshine duration. Fourth, the activity pattern of the population would affect the personal level of ozone exposure. Season and weather, especially temperature (45–47) were significantly associated with the activity patterns of the population. It was also projected that climate change would influence physical activity differently over regions (48). With a generally lower concentration of ozone indoor (49), the exposure to ozone may differ at the individual level, resulting in distinct cardiovascular risks. In China, most of population has already retired for several years at the age of 65 and thus, has no obligations to commute. Shenzhen has two respective warning systems for high air pollution and extreme weather condition, timely advising vulnerable population with underlying health conditions to avoid outside activities. Furthermore, air conditioning systems are widely covered in Shenzhen. Therefore, individual exposure of senior population in our research might be low. This may explain that younger population was found to be more susceptible in our research. Therefore, future research accessing the individual exposure to ozone may help to further explain the differences between regions.

Previous evidence suggested several plausible mechanisms regarding the impacts of ozone, and though insufficient, some studies considered the modification of temperature on ozone-related cardiovascular events. Inflammation was one of the most identified pathways explaining the cardiovascular effects of ozone. Exposure to ozone was revealed to be associated with systematic inflammation (50, 51) as well as pulmonary inflammation (52, 53). Impair of pulmonary function has been causally related to cardiovascular events through inflammation and increasing fibrinogen in lungs (54, 55). Therefore, there were scholars considering the indices of lung function when they explored the cardiovascular effects of ozone. Though one study in healthy adults reported no significant changes of lung function under the exposure of ozone (52), more investigations revealed its adverse impacts on ventilation function (56–59).

Another interpretation about the adverse effects of ozone on CVD-caused mortality was that ozone influenced the process of hemostasis and thrombosis. Plasminogen activator inhibitor-1 (PAI-1), which could contain fibrinolysis, was found to increase with the exposure of ozone (51) and thus might result in the formation of thrombi. Further, such changes became more significant when combined with heat (60, 61). Ozone was also underlined to elevate the risk of thrombosis through platelet activation (52). Besides, it was observed that high-density lipoprotein cholesterol (HDL-C) increased when exposed to ozone only (61) and exposed to both ozone and heat (60). In addition, low-density lipoprotein cholesterol (LDL-C) and total cholesterol decreased under combined exposure to ozone and heat (60). These alterations of lipoproteins could result in thrombosis as well.

Previous research reported the changes of blood pressure when exposed to ozone, yet the findings were inconsistent. Increasing blood pressure related to ozone was discovered among healthy adults, while the same research reported decreased arterial stiffness marker which was positively related to blood pressure (52). Additionally, other research in animal and human with certain underlying medical conditions noted decreased blood pressure, heart rate, and artery elasticity after ozone exposure, denoting the impacts of ozone on parasympathetic nervous system and endothelial function (51, 56, 62). This could be one of the possible reasons about the negative effects of ozone on mortality due to hypertensive diseases observed in our research.

A few limitations should be noted while interpreting our findings. First, the sociodemographic information and the underlying health conditions of population were absent due to the limitation of data. Second, our analyses were based on the level of outdoor ozone. Due to the differences of mobility modes among groups of population in the days with high temperature and extreme heat, the individual exposure to ozone may vary and thus affect our results. Third, the current-day levels of other pollutants and meteorological factors were employed in our research, and hence, the effects may be underestimated.

## 5. Conclusion

In conclusion, our findings indicated the short-term cardiovascular impacts of ozone below the current national standard of air quality, which suggested an improved national standard, and more interventions reducing ambient ozone pollution in China. Compared to season, temperature was a more sensitive modifier for short-term ozone-associated effects on cardiovascular health. High temperature, especially extreme heat, could significantly enhance the adverse effects of ozone on cardiovascular mortality in males and the population under 65 years old. Thus, it is also recommended to promote an adjusted system of early warning and emergency response considering both temperature and air pollution.

## Data availability statement

The data analyzed in this study is subject to the following licenses/restrictions: The data that support the findings of this study are available from China National Environmental Monitoring Centre and Shenzhen Center for Disease Control and Prevention but restrictions apply to the availability of these data, which were used under license for the current study, and so are not publicly available. Data are however available from the authors upon reasonable request and with permission of China National Environmental Monitoring Centre and Shenzhen Center for Disease Control and Prevention. Requests to access these datasets should be directed to FZ, zhangfy@cnemc.cn.

## Author contributions

PG analyzed and interpreted the data, and drafted the manuscript. YW and YF curated the mortality data. LH, JC, and FZ curated the data of environmental variables. LW acquired the funding. JC, FZ, TK, and PM supervised the conduction of this research and contributed to the final manuscript. All authors read and approved the final manuscript.

## Funding

This study was supported by National Key R&D Plan (2022YFC3702604); Panjun Gao was personally funded by China Scholarship Council (201907720108). The sponsors had no roles in study design, in the collection, analysis and interpretation of data, in the writing of the report, or in the decision to submit the article for publication.

## Conflict of interest

The authors declare that the research was conducted in the absence of any commercial or financial relationships that could be construed as a potential conflict of interest.

## Publisher’s note

All claims expressed in this article are solely those of the authors and do not necessarily represent those of their affiliated organizations, or those of the publisher, the editors and the reviewers. Any product that may be evaluated in this article, or claim that may be made by its manufacturer, is not guaranteed or endorsed by the publisher.

## References

[1] Zhang JJ Wei Y Fang Z. Ozone pollution: a major health hazard worldwide. Front Immunol. (2019) 10:2518. doi: 10.3389/fimmu.2019.02518, PMID: 31736954 PMC6834528

[2] Vicedo-Cabrera AM Sera F Liu C Armstrong B Milojevic A Guo Y. Short term association between ozone and mortality: global two stage time series study in 406 locations in 20 countries. BMJ. (2020) 368:m108. doi: 10.1136/bmj.m108, PMID: 32041707 PMC7190035

[3] Anenberg SC Horowitz LW Tong DQ West JJ. An estimate of the global burden of anthropogenic ozone and fine particulate matter on premature human mortality using atmospheric modeling. Environ Health Perspect. (2010) 118:1189–95. doi: 10.1289/ehp.0901220, PMID: 20382579 PMC2944076

[4] Wong TW Lau TS Yu TS Neller A Wong SL Tam W. Air pollution and hospital admissions for respiratory and cardiovascular diseases in Hong Kong. Occup Environ Med. (1999) 56:679–83. doi: 10.1136/oem.56.10.679, PMID: 10658547 PMC1757671

[5] Ballester F Rodriguez P Iniguez C Saez M Daponte A Galan I. Air pollution and cardiovascular admissions association in Spain: results within the EMECAS project. J Epidemiol Community Health. (2006) 60:328–36. doi: 10.1136/jech.2005.037978, PMID: 16537350 PMC2566168

[6] Fischer P Hoek G Brunekreef B Verhoeff A van Wijnen J. Air pollution and mortality in the Netherlands: are the elderly more at risk? Eur Respir J Suppl. (2003) 40:34S–8S. doi: 10.1183/09031936.03.00402503, PMID: 12762572

[7] Khaniabadi YO Hopke PK Goudarzi G Daryanoosh SM Jourvand M Basiri H. Cardiopulmonary mortality and COPD attributed to ambient ozone. Environ Res. (2017) 152:336–41. doi: 10.1016/j.envres.2016.10.008, PMID: 27842286

[8] Revich B Shaposhnikov D. The effects of particulate and ozone pollution on mortality in Moscow. Russia Air Qual Atmos Health. (2010) 3:117–23. doi: 10.1007/s11869-009-0058-7, PMID: 20495603 PMC2860096

[9] Klompmaker JO Hart JE James P Sabath MB Wu X Zanobetti A. Air pollution and cardiovascular disease hospitalization - are associations modified by greenness, temperature and humidity? Environ Int. (2021) 156:106715. doi: 10.1016/j.envint.2021.106715, PMID: 34218186 PMC8380672

[10] Pascal M Wagner V Chatignoux E Falq G Corso M Blanchard M. Ozone and short-term mortality in nine French cities: influence of temperature and season. Atmos Environ. (2012) 62:566–72. doi: 10.1016/j.atmosenv.2012.09.009

[11] Halonen JI Lanki T Tiittanen P Niemi JV Loh M Pekkanen J. Ozone and cause-specific cardiorespiratory morbidity and mortality. J Epidemiol Community Health. (2010) 64:814–20. doi: 10.1136/jech.2009.087106, PMID: 19854743

[12] Hanna AF Yeatts KB Xiu A Zhu Z Smith RL Davis NN. Associations between ozone and morbidity using the spatial synoptic classification system. Environ Health. (2011) 10:49. doi: 10.1186/1476-069X-10-49, PMID: 21609456 PMC3117763

[13] Nuvolone D Balzi D Pepe P Chini M Scala D Giovannini F. Ozone short-term exposure and acute coronary events: a multicities study in Tuscany (Italy). Environ Res. (2013) 126:17–23. doi: 10.1016/j.envres.2013.08.002, PMID: 24011457

[14] Koken PJM Piver WT Ye F Elixhauser A Olsen LM Portier CJ. Temperature, air pollution, and hospitalization for cardiovascular diseases among elderly people in Denver. Environ Health Perspect. (2003) 111:1312–7. doi: 10.1289/ehp.5957, PMID: 12896852 PMC1241612

[15] Mazidi M Speakman JR. Impact of obesity and ozone on the association between particulate air pollution and cardiovascular disease and stroke mortality among US adults. J Am Heart Assoc. (2018) 7:e008006. doi: 10.1161/JAHA.117.008006, PMID: 29848499 PMC6015356

[16] Yin P Chen R Wang L Meng X Liu C Niu Y. Ambient ozone pollution and daily mortality: a nationwide study in 272 Chinese cities. Environ Health Perspect. (2017) 125:117006. doi: 10.1289/EHP1849, PMID: 29212061 PMC5947936

[17] Li K Jacob DJ Shen L Lu X De Smedt I Liao H. Increases in surface ozone pollution in China from 2013 to 2019: anthropogenic and meteorological influences. Atmos Chem Phys. (2020) 20:11423–33. doi: 10.5194/acp-20-11423-2020

[18] Lu X Hong J Zhang L Cooper OR Schultz MG Xu X. Severe surface ozone pollution in China: a global perspective. Environ Sci Technol Lett. (2018) 5:487–94. doi: 10.1021/acs.estlett.8b00366

[19] Hu C Kang P Jaffe DA Li C Zhang X Wu K. Understanding the impact of meteorology on ozone in 334 cities of China. Atmos Environ. (2021) 248:118221. doi: 10.1016/j.atmosenv.2021.118221

[20] Orru H Andersson C Ebi KL Langner J Astrom C Forsberg B. Impact of climate change on ozone-related mortality and morbidity in Europe. Eur Respir J. (2013) 41:285–94. doi: 10.1183/09031936.00210411, PMID: 22743679

[21] Li J Yin P Wang L Zhang X Liu J Liu Y. Ambient ozone pollution and years of life lost: association, effect modification, and additional life gain from a nationwide analysis in China. Environ Int. (2020) 141:105771. doi: 10.1016/j.envint.2020.105771, PMID: 32402982

[22] Sun Q Wang W Chen C Ban J Xu D Zhu P. Acute effect of multiple ozone metrics on mortality by season in 34 Chinese counties in 2013-2015. J Intern Med. (2018) 283:481–8. doi: 10.1111/joim.12724, PMID: 29247470 PMC6764438

[23] Zhang J Gao Y Luo K Leung LR Zhang Y Wang K. Impacts of compound extreme weather events on ozone in the present and future. Atmos Chem Phys. (2018) 18:9861–77. doi: 10.5194/acp-18-9861-2018

[24] Pu X Wang TJ Huang X Melas D Zanis P Papanastasiou DK. Enhanced surface ozone during the heat wave of 2013 in Yangtze River Delta region. China Sci Total Environ. (2017) 603-604:807–16. doi: 10.1016/j.scitotenv.2017.03.056, PMID: 28442137

[25] Lin X Yuan Z Yang L Luo H Li W. Impact of extreme meteorological events on ozone in the Pearl River Delta. China Aerosol and Air Quality Research. (2019) 19:1307–24. doi: 10.4209/aaqr.2019.01.0027

[26] Lu R-Y Chen R-D. A review of recent studies on extreme heat in China. Atmospheric Oceanic Sci Lett. (2016) 9:114–21. doi: 10.1080/16742834.2016.1133071

[27] Wang T Xue L Brimblecombe P Lam YF Li L Zhang L. Ozone pollution in China: a review of concentrations, meteorological influences, chemical precursors, and effects. Sci Total Environ. (2017) 575:1582–96. doi: 10.1016/j.scitotenv.2016.10.081, PMID: 27789078

[28] Wang L Bai Y Zhang F Wang W Liu X Krafft T. Spatiotemporal patterns of ozone and cardiovascular and respiratory disease mortalities due to ozone in Shenzhen. Sustainability. (2017) 9:559. doi: 10.3390/su9040559

[29] Figueiras A Domenech-Massons JM Cadarso C. Regression models: calculating the confidence interval of effects in the presence of interactions. Stat Med. (1998) 17:2099–105. doi: 10.1002/(SICI)1097-0258(19980930)17:18<2099::AID-SIM905>3.0.CO;2-6 9789916

[30] World Health Organization. WHO global air quality guidelines. Particulate matter (PM2.5 and PM10), ozone, nitrogen dioxide, sulfur dioxide and carbon monoxide. Geneva: World Health Organization (2021). 34662007

[31] Dueñas C Fernández MC Cañete S Carretero J Liger E. Assessment of ozone variations and meteorological effects in an urban area in the Mediterranean coast. Sci Total Environ. (2002) 299:97–113. doi: 10.1016/S0048-9697(02)00251-6, PMID: 12462577

[32] Zhang F Liu X Zhou L Yu Y Wang L Lu J. Spatiotemporal patterns of particulate matter (PM) and associations between PM and mortality in Shenzhen. China BMC Public Health. (2016) 16:215. doi: 10.1186/s12889-016-2725-6, PMID: 26935584 PMC4776388

[33] Hunova I Maly M Rezacova J Branis M. Association between ambient ozone and health outcomes in Prague. Int Arch Occup Environ Health. (2013) 86:89–97. doi: 10.1007/s00420-012-0751-y, PMID: 22366988

[34] Sui X Zhang J Zhang Q Sun S Lei R Zhang C. The short-term effect of PM2.5/O3 on daily mortality from 2013 to 2018 in Hefei, China. Environ Geochem Health. (2021) 43:153–69. doi: 10.1007/s10653-020-00689-x, PMID: 32785823

[35] Zhang Y Huang W London SJ Song G Chen G Jiang L. Ozone and daily mortality in Shanghai, China. Environ Health Perspect. (2006) 114:1227–32. doi: 10.1289/ehp.9014, PMID: 16882530 PMC1552011

[36] de Almeida SP Casimiro E Calheiros J. Short-term association between exposure to ozone and mortality in Oporto. Portugal Environ Res. (2011) 111:406–10. doi: 10.1016/j.envres.2011.01.024, PMID: 21315327

[37] Ng CF Ueda K Nitta H Takeuchi A. Seasonal variation in the acute effects of ozone on premature mortality among elderly Japanese. Environ Monit Assess. (2013) 185:8767–76. doi: 10.1007/s10661-013-3211-6, PMID: 23604788

[38] Li Y Shang Y Zheng C Ma Z. Estimated acute effects of ozone on mortality in a Rural District of Beijing, China, 2005(−)2013: a time-stratified Case-crossover study. Int J Environ Res Public Health. (2018) 15:2460. doi: 10.3390/ijerph15112460 30400565 PMC6266742

[39] Gryparis A Forsberg B Katsouyanni K Analitis A Touloumi G Schwartz J. Acute effects of ozone on mortality from the "air pollution and health: a European approach" project. Am J Respir Crit Care Med. (2004) 170:1080–7. doi: 10.1164/rccm.200403-333OC 15282198

[40] Qin L Gu J Liang S Fang F Bai W Liu X. Seasonal association between ambient ozone and mortality in Zhengzhou. China Int J Biometeorol. (2017) 61:1003–10. doi: 10.1007/s00484-016-1279-8, PMID: 27981338

[41] Goudarzi G Geravandi S Foruozandeh H Babaei AA Alavi N Niri MV. Cardiovascular and respiratory mortality attributed to ground-level ozone in Ahvaz, Iran. Environ Monit Assess. (2015) 187:487. doi: 10.1007/s10661-015-4674-4, PMID: 26141926

[42] Shi W Sun Q Du P Tang S Chen C Sun Z. Modification effects of temperature on the ozone-mortality relationship: a nationwide multicounty study in China. Environ Sci Technol. (2020) 54:2859–68. doi: 10.1021/acs.est.9b05978, PMID: 32022552

[43] Chen K Wolf K Breitner S Gasparrini A Stafoggia M Samoli E. Two-way effect modifications of air pollution and air temperature on total natural and cardiovascular mortality in eight European urban areas. Environ Int. (2018) 116:186–96. doi: 10.1016/j.envint.2018.04.021, PMID: 29689465

[44] Ren C Williams GM Mengersen K Morawska L Tong S. Temperature enhanced effects of ozone on cardiovascular mortality in 95 large US communities, 1987-2000: assessment using the NMMAPS data. Arch Environ Occup Health. (2009) 64:177–84. doi: 10.1080/19338240903240749, PMID: 19864220

[45] Horanont T Phithakkitnukoon S Leong TW Sekimoto Y Shibasaki R. Weather effects on the patterns of people’s everyday activities: a study using GPS traces of mobile phone users. PLoS One. (2013) 8:e81153. doi: 10.1371/journal.pone.0081153, PMID: 24367481 PMC3867318

[46] Turrisi TB Bittel KM West AB Hojjatinia S Hojjatinia S Mama SK. Seasons, weather, and device-measured movement behaviors: a scoping review from 2006 to 2020. Int J Behav Nutr Phys Act. (2021) 18:24. doi: 10.1186/s12966-021-01091-1, PMID: 33541375 PMC7863471

[47] McCurdy T Graham SE. Using human activity data in exposure models: analysis of discriminating factors. J Expo Anal Environ Epidemiol. (2003) 13:294–317. doi: 10.1038/sj.jea.7500281, PMID: 12923556

[48] Obradovich N Fowler JH. Climate change may alter human physical activity patterns. Nature human. Behaviour. (2017) 1:0097. doi: 10.1038/s41562-017-0097

[49] Salonen H Salthammer T Morawska L. Human exposure to ozone in school and office indoor environments. Environ Int. (2018) 119:503–14. doi: 10.1016/j.envint.2018.07.012, PMID: 30053738

[50] Arjomandi M Wong H Donde A Frelinger J Dalton S Ching W. Exposure to medium and high ambient levels of ozone causes adverse systemic inflammatory and cardiac autonomic effects. Am J Physiol Heart Circ Physiol. (2015) 308:H1499–509. doi: 10.1152/ajpheart.00849.2014, PMID: 25862833 PMC4469877

[51] Mirowsky JE Carraway MS Dhingra R Tong H Neas L Diaz-Sanchez D. Ozone exposure is associated with acute changes in inflammation, fibrinolysis, and endothelial cell function in coronary artery disease patients. Environ Health. (2017) 16:126. doi: 10.1186/s12940-017-0335-0, PMID: 29157250 PMC5697214

[52] Day DB Xiang J Mo J Li F Chung M Gong J. Association of ozone exposure with cardiorespiratory pathophysiologic mechanisms in healthy adults. JAMA Intern Med. (2017) 177:1344–53. doi: 10.1001/jamainternmed.2017.2842, PMID: 28715576 PMC5710579

[53] Ramot Y Kodavanti UP Kissling GE Ledbetter AD Nyska A. Clinical and pathological manifestations of cardiovascular disease in rat models: the influence of acute ozone exposure. Inhal Toxicol. (2015) 27:26–38. doi: 10.3109/08958378.2014.954168, PMID: 26667329

[54] Cheng YJ Chen ZG Li ZY Mei WY Bi WT Luo DL. Longitudinal change in lung function and subsequent risks of cardiovascular events: evidence from four prospective cohort studies. BMC Med. (2021) 19:153. doi: 10.1186/s12916-021-02023-3, PMID: 34210292 PMC8252272

[55] Sin DD Wu L Man SF. The relationship between reduced lung function and cardiovascular mortality: a population-based study and a systematic review of the literature. Chest. (2005) 127:1952–9. doi: 10.1378/chest.127.6.1952 15947307

[56] Watkinson WP Campen MJ Nolan JP Costa DL. Cardiovascular and systemic responses to inhaled pollutants in rodents: effects of ozone and particulate matter. Environ Health Perspect. (2001) 109:539–46. doi: 10.1289/ehp.01109s4539, PMID: 11544160 PMC1240578

[57] Gong H Jr Wong R Sarma RJ Linn WS Sullivan ED Shamoo DA. Cardiovascular effects of ozone exposure in human volunteers. Am J Respir Crit Care Med. (1998) 158:538–46. doi: 10.1164/ajrccm.158.2.9709034 9700133

[58] Devlin RB Duncan KE Jardim M Schmitt MT Rappold AG Diaz-Sanchez D. Controlled exposure of healthy young volunteers to ozone causes cardiovascular effects. Circulation. (2012) 126:104–11. doi: 10.1161/CIRCULATIONAHA.112.094359, PMID: 22732313

[59] Dye JA Ledbetter AD Schladweiler MC Costa DL Kodavanti UP. Whole body plethysmography reveals differential ventilatory responses to ozone in rat models of cardiovascular disease. Inhal Toxicol. (2015) 27:14–25. doi: 10.3109/08958378.2014.954167, PMID: 26667328

[60] Song QQ Niu JP Zhang SY Liang TT Zhou J Feng SS. Effects of simulated heat wave and ozone on high fat diet ApoE deficient mice. Biomed Environ Sci. (2018) 31:757–68. doi: 10.3967/bes2018.101, PMID: 30423277

[61] Liang T Niu J Zhang S Song Q Zhou J. Effects of high-temperature heat wave and ozone on hypertensive rats. Int J Biometeorol. (2020) 64:1039–50. doi: 10.1007/s00484-019-01788-w, PMID: 32440829

[62] Hoffmann B Luttmann-Gibson H Cohen A Zanobetti A de Souza C Foley C. Opposing effects of particle pollution, ozone, and ambient temperature on arterial blood pressure. Environ Health Perspect. (2012) 120:241–6. doi: 10.1289/ehp.1103647, PMID: 22020729 PMC3279434

