# Supplementary material for: Adverse short-term effects of ozone on cardiovascular mortalities modified by season and temperature: a time-series study
Source: Front Public Health. 2023 Jun 9;11:1182337. doi: 10.3389/fpubh.2023.1182337 (PMC10288843; doi:10.3389/fpubh.2023.1182337)
Supplement: Supplementary file 1 [file Table_1.DOCX]

| Table S1 Summary of ozone and meteorological factors | | | | | | | | | |
| --- | --- | --- | --- | --- | --- | --- | --- | --- | --- |
|  |  |  | Mean | SD | Minimum | 25% | Median | 75% | Maximum |
| O_3_-1h(μg/m^3^) | ns |  | 98.4 | 42.8 | 18.0 | 65.0 | 91.0 | 123.0 | 290.0 |
|  | cold season | | 96.5 | 33.7 | 18.0 | 72.0 | 94.0 | 119.0 | 223.0 |
|  | warm season | | 100.2 | 50.1 | 28.0 | 60.0 | 86.0 | 132.0 | 290.0 |
|  | low temperature | | 98.8 | 34.1 | 18.0 | 74.0 | 98.0 | 121.0 | 223.0 |
|  | high temperature | | 97.9 | 50.2 | 28.0 | 59.0 | 81.0 | 128.0 | 290.0 |
|  | extreme heat | | 144.8 | 58.8 | 55.0 | 91.0 | 137.0 | 189.5 | 287.0 |
| O_3_-8h(μg/m^3^) | ns |  | 81.1 | 36.3 | 14.0 | 52.0 | 75.0 | 103.0 | 246.0 |
|  | cold season | | 80.1 | 29.1 | 14.0 | 58.0 | 80.0 | 101.0 | 186.0 |
|  | warm season | | 82.0 | 42.2 | 21.0 | 48.8 | 69.0 | 109.3 | 246.0 |
|  | low temperature | | 82.5 | 29.8 | 14.0 | 60.0 | 82.0 | 103.0 | 186.0 |
|  | high temperature | | 79.7 | 41.9 | 21.0 | 47.0 | 65.0 | 105.0 | 246.0 |
|  | extreme heat | | 116.1 | 47.9 | 46.0 | 73.0 | 110.0 | 150.5 | 237.0 |
| Tem (℃) | ns |  | 23.5 | 5.4 | 3.5 | 19.5 | 24.8 | 28.1 | 33.0 |
|  | cold season | | 19.2 | 4.1 | 3.5 | 16.5 | 19.5 | 22.4 | 28.0 |
|  | warm season | | 27.7 | 2.1 | 18.8 | 26.5 | 28.1 | 29.4 | 33.0 |
|  | low temperature | | 19.1 | 3.9 | 3.5 | 16.6 | 19.6 | 22.4 | 24.8 |
|  | high temperature | | 28.0 | 1.6 | 24.9 | 26.7 | 28.1 | 29.4 | 33.0 |
|  | extreme heat | | 30.8 | 0.5 | 30.4 | 30.5 | 30.7 | 31.0 | 33.0 |
| Hum (%) | ns |  | 75.6 | 13.1 | 19.0 | 69.5 | 78.0 | 84.5 | 100.0 |
|  | cold season | | 72.6 | 14.8 | 19.0 | 65.0 | 75.5 | 83.3 | 100.0 |
|  | warm season | | 78.5 | 10.4 | 34.0 | 73.0 | 79.4 | 85.8 | 99.5 |
|  | low temperature | | 72.2 | 15.2 | 19.0 | 64.3 | 75.0 | 83.3 | 100.0 |
|  | high temperature | | 79.0 | 9.3 | 38.8 | 73.5 | 79.8 | 85.3 | 99.5 |
|  | extreme heat | | 71.8 | 7.8 | 51.0 | 68.0 | 72.3 | 76.0 | 90.3 |
| Pre (mm) | ns |  | 5.3 | 15.9 | 0.0 | 0.0 | 0.0 | 1.3 | 187.8 |
|  | cold season | | 2.2 | 8.5 | 0.0 | 0.0 | 0.0 | 0.0 | 153.8 |
|  | warm season | | 8.4 | 20.4 | 0.0 | 0.0 | 0.0 | 6.0 | 187.8 |
|  | low temperature | | 2.9 | 11.8 | 0.0 | 0.0 | 0.0 | 0.1 | 187.8 |
|  | high temperature | | 7.8 | 19.0 | 0.0 | 0.0 | 0.0 | 5.4 | 173.5 |
|  | extreme heat | | 1.0 | 3.7 | 0.0 | 0.0 | 0.0 | 0.0 | 17.1 |
| BP (hPa) | ns |  | 1005.5 | 6.5 | 983.1 | 1000.7 | 1005.5 | 1010.4 | 1027.2 |
|  | cold season | | 1010.3 | 4.3 | 995.4 | 1007.2 | 1010.2 | 1013.3 | 1027.2 |
|  | warm season | | 1000.8 | 4.5 | 983.1 | 997.9 | 1000.8 | 1003.4 | 1013.7 |
|  | low temperature | | 1010.3 | 4.3 | 994.4 | 1007.4 | 1010.3 | 1013.3 | 1027.2 |
|  | high temperature | | 1000.5 | 4.2 | 983.1 | 997.8 | 1000.7 | 1003.1 | 1012.8 |
|  | extreme heat | | 996.6 | 3.8 | 989.3 | 993.9 | 995.7 | 1000.0 | 1003.7 |
| WS (m/s) | ns |  | 2.0 | 0.7 | 0.4 | 1.4 | 1.8 | 2.3 | 6.1 |
|  | cold season | | 2.0 | 0.7 | 0.6 | 1.5 | 1.9 | 2.4 | 5.4 |
|  | warm season | | 1.9 | 0.7 | 0.4 | 1.4 | 1.8 | 2.2 | 6.1 |
|  | low temperature | | 2.0 | 0.7 | 0.6 | 1.5 | 2.0 | 2.4 | 5.9 |
|  | high temperature | | 1.9 | 0.7 | 0.4 | 1.4 | 1.8 | 2.2 | 6.1 |
|  | extreme heat | | 1.9 | 0.7 | 0.8 | 1.4 | 1.8 | 2.4 | 3.7 |
| SSD (h) | ns |  | 5.2 | 3.8 | 0.0 | 1.4 | 5.5 | 8.7 | 12.5 |
|  | cold season | | 4.4 | 3.7 | 0.0 | 0.5 | 4.0 | 7.9 | 11.3 |
|  | warm season | | 6.1 | 3.8 | 0.0 | 2.7 | 6.7 | 9.5 | 12.5 |
|  | low temperature | | 4.2 | 3.7 | 0.0 | 0.3 | 3.6 | 7.9 | 10.7 |
|  | high temperature | | 6.3 | 3.6 | 0.0 | 3.3 | 6.9 | 9.5 | 12.5 |
|  | extreme heat | | 9.6 | 1.6 | 4.6 | 8.6 | 9.7 | 10.8 | 12.1 |
