# Supplementary material for: Adverse short-term effects of ozone on cardiovascular mortalities modified by season and temperature: a time-series study
Source: Front Public Health. 2023 Jun 9;11:1182337. doi: 10.3389/fpubh.2023.1182337 (PMC10288843; doi:10.3389/fpubh.2023.1182337)
Supplement: Supplementary file 2 [file Table_2.docx]

| Table S2 Summary of cardiovascular death causes | | | | | | | | | |
| --- | --- | --- | --- | --- | --- | --- | --- | --- | --- |
|  |  |  | Mean | SD | Minimum | 25% | Median | 75% | Maximum |
| Total | CVD | ns | 9.9 | 5.4 | 0 | 5 | 9 | 14 | 33 |
|  |  | cold season | 10.7 | 5.8 | 0 | 6 | 10 | 15 | 33 |
|  |  | warm season | 9.0 | 4.8 | 0 | 5 | 9 | 13 | 24 |
|  |  | low temperature | 10.7 | 5.8 | 0 | 6 | 10 | 15 | 33 |
|  |  | high temperature | 9.0 | 4.8 | 0 | 5 | 9 | 13 | 24 |
|  |  | extreme heat | 9.6 | 5.2 | 1 | 5 | 9 | 13 | 21 |
|  | HBP | ns | 0.9 | 1.0 | 0 | 0 | 1 | 1 | 6 |
|  |  | cold season | 1.0 | 1.0 | 0 | 0 | 1 | 2 | 6 |
|  |  | warm season | 0.8 | 0.9 | 0 | 0 | 1 | 1 | 5 |
|  |  | low temperature | 1.0 | 1.0 | 0 | 0 | 1 | 2 | 6 |
|  |  | high temperature | 0.8 | 0.9 | 0 | 0 | 1 | 1 | 5 |
|  |  | extreme heat | 0.8 | 1.2 | 0 | 0 | 0 | 1 | 5 |
|  | IHD | ns | 4.0 | 3.0 | 0 | 2 | 3 | 6 | 17 |
|  |  | cold season | 4.3 | 3.1 | 0 | 2 | 4 | 6 | 17 |
|  |  | warm season | 3.6 | 2.7 | 0 | 1 | 3 | 5 | 13 |
|  |  | low temperature | 4.2 | 3.1 | 0 | 2 | 4 | 6 | 17 |
|  |  | high temperature | 3.7 | 2.7 | 0 | 1 | 3 | 6 | 13 |
|  |  | extreme heat | 3.8 | 2.8 | 0 | 1 | 3 | 6 | 11 |
|  | STR | ns | 3.6 | 2.4 | 0 | 2 | 3 | 5 | 14 |
|  |  | cold season | 3.9 | 2.6 | 0 | 2 | 4 | 6 | 14 |
|  |  | warm season | 3.2 | 2.1 | 0 | 2 | 3 | 5 | 11 |
|  |  | low temperature | 3.9 | 2.6 | 0 | 2 | 4 | 5.25 | 14 |
|  |  | high temperature | 3.2 | 2.1 | 0 | 2 | 3 | 5 | 11 |
|  |  | extreme heat | 3.7 | 2.2 | 0 | 2 | 4 | 5 | 10 |
| Female | CVD | ns | 3.7 | 2.5 | 0 | 2 | 3 | 5 | 16 |
|  |  | cold season | 4.0 | 2.7 | 0 | 2 | 4 | 6 | 16 |
|  |  | warm season | 3.3 | 2.3 | 0 | 2 | 3 | 5 | 13 |
|  |  | low temperature | 4.0 | 2.7 | 0 | 2 | 4 | 6 | 16 |
|  |  | high temperature | 3.3 | 2.3 | 0 | 2 | 3 | 5 | 12 |
|  |  | extreme heat | 3.6 | 2.6 | 0 | 2 | 3 | 5 | 10 |
|  | HBP | ns | 0.4 | 0.6 | 0 | 0 | 0 | 1 | 5 |
|  |  | cold season | 0.4 | 0.6 | 0 | 0 | 0 | 1 | 5 |
|  |  | warm season | 0.3 | 0.6 | 0 | 0 | 0 | 1 | 3 |
|  |  | low temperature | 0.4 | 0.6 | 0 | 0 | 0 | 1 | 5 |
|  |  | high temperature | 0.3 | 0.5 | 0 | 0 | 0 | 1 | 3 |
|  |  | extreme heat | 0.4 | 0.7 | 0 | 0 | 0 | 1 | 3 |
|  | IHD | ns | 1.4 | 1.5 | 0 | 0 | 1 | 2 | 8 |
|  |  | cold season | 1.6 | 1.6 | 0 | 0 | 1 | 2 | 8 |
|  |  | warm season | 1.3 | 1.3 | 0 | 0 | 1 | 2 | 8 |
|  |  | low temperature | 1.5 | 1.5 | 0 | 0 | 1 | 2 | 8 |
|  |  | high temperature | 1.3 | 1.3 | 0 | 0 | 1 | 2 | 8 |
|  |  | extreme heat | 1.3 | 1.4 | 0 | 0 | 1 | 2 | 6 |
|  | STR | ns | 1.4 | 1.3 | 0 | 0 | 1 | 2 | 7 |
|  |  | cold season | 1.5 | 1.4 | 0 | 0 | 1 | 2 | 7 |
|  |  | warm season | 1.3 | 1.2 | 0 | 0 | 1 | 2 | 6 |
|  |  | low temperature | 1.5 | 1.4 | 0 | 0 | 1 | 2 | 7 |
|  |  | high temperature | 1.3 | 1.2 | 0 | 0 | 1 | 2 | 6 |
|  |  | extreme heat | 1.4 | 1.3 | 0 | 0 | 1 | 2 | 5 |
| Male | CVD | ns | 6.2 | 3.7 | 0 | 3 | 6 | 9 | 24 |
|  |  | cold season | 6.7 | 4.0 | 0 | 4 | 6 | 9 | 24 |
|  |  | warm season | 5.7 | 3.3 | 0 | 3 | 5 | 8 | 17 |
|  |  | low temperature | 6.7 | 3.9 | 0 | 4 | 6 | 9 | 24 |
|  |  | high temperature | 5.7 | 3.4 | 0 | 3 | 5 | 8 | 17 |
|  |  | extreme heat | 6.0 | 3.5 | 1 | 3 | 5 | 8.5 | 13 |
|  | HBP | ns | 0.5 | 0.7 | 0 | 0 | 0 | 1 | 4 |
|  |  | cold season | 0.6 | 0.8 | 0 | 0 | 0 | 1 | 4 |
|  |  | warm season | 0.5 | 0.7 | 0 | 0 | 0 | 1 | 4 |
|  |  | low temperature | 0.6 | 0.8 | 0 | 0 | 0 | 1 | 4 |
|  |  | high temperature | 0.5 | 0.7 | 0 | 0 | 0 | 1 | 4 |
|  |  | extreme heat | 0.4 | 0.7 | 0 | 0 | 0 | 1 | 2 |
|  | IHD | ns | 2.5 | 2.1 | 0 | 1 | 2 | 4 | 11 |
|  |  | cold season | 2.7 | 2.2 | 0 | 1 | 2 | 4 | 11 |
|  |  | warm season | 2.4 | 2.0 | 0 | 1 | 2 | 4 | 9 |
|  |  | low temperature | 2.7 | 2.2 | 0 | 1 | 2 | 4 | 11 |
|  |  | high temperature | 2.4 | 2.0 | 0 | 1 | 2 | 4 | 10 |
|  |  | extreme heat | 2.4 | 2.1 | 0 | 1 | 2 | 4 | 7 |
|  | STR | ns | 2.2 | 1.7 | 0 | 1 | 2 | 3 | 10 |
|  |  | cold season | 2.4 | 1.8 | 0 | 1 | 2 | 3 | 10 |
|  |  | warm season | 2.0 | 1.5 | 0 | 1 | 2 | 3 | 7 |
|  |  | low temperature | 2.4 | 1.8 | 0 | 1 | 2 | 3 | 10 |
|  |  | high temperature | 2.0 | 1.5 | 0 | 1 | 2 | 3 | 8 |
|  |  | extreme heat | 2.3 | 1.5 | 0 | 1 | 2 | 3 | 6 |
| Senior | CVD | ns | 5.9 | 3.8 | 0 | 3 | 5 | 8 | 24 |
|  |  | cold season | 6.6 | 4.1 | 0 | 3 | 6 | 9 | 24 |
|  |  | warm season | 5.3 | 3.3 | 0 | 3 | 5 | 8 | 18 |
|  |  | low temperature | 6.5 | 4.0 | 0 | 3 | 6 | 9 | 24 |
|  |  | high temperature | 5.3 | 3.4 | 0 | 3 | 5 | 8 | 19 |
|  |  | extreme heat | 5.6 | 3.6 | 0 | 3 | 5 | 8 | 16 |
|  | HBP | ns | 0.5 | 0.8 | 0 | 0 | 0 | 1 | 5 |
|  |  | cold season | 0.6 | 0.8 | 0 | 0 | 0 | 1 | 5 |
|  |  | warm season | 0.5 | 0.7 | 0 | 0 | 0 | 1 | 5 |
|  |  | low temperature | 0.6 | 0.8 | 0 | 0 | 0 | 1 | 5 |
|  |  | high temperature | 0.5 | 0.7 | 0 | 0 | 0 | 1 | 5 |
|  |  | extreme heat | 0.5 | 0.8 | 0 | 0 | 0 | 1 | 3 |
|  | IHD | ns | 2.5 | 2.1 | 0 | 1 | 2 | 4 | 15 |
|  |  | cold season | 2.8 | 2.2 | 0 | 1 | 2 | 4 | 15 |
|  |  | warm season | 2.2 | 1.9 | 0 | 1 | 2 | 3 | 11 |
|  |  | low temperature | 2.8 | 2.2 | 0 | 1 | 2 | 4 | 15 |
|  |  | high temperature | 2.3 | 1.9 | 0 | 1 | 2 | 3 | 11 |
|  |  | extreme heat | 2.3 | 1.9 | 0 | 1 | 2 | 3 | 7 |
|  | STR | ns | 2.3 | 1.9 | 0 | 1 | 2 | 3 | 11 |
|  |  | cold season | 2.6 | 2.1 | 0 | 1 | 2 | 4 | 11 |
|  |  | warm season | 2.1 | 1.7 | 0 | 1 | 2 | 3 | 9 |
|  |  | low temperature | 2.5 | 2.0 | 0 | 1 | 2 | 4 | 11 |
|  |  | high temperature | 2.1 | 1.7 | 0 | 1 | 2 | 3 | 10 |
|  |  | extreme heat | 2.4 | 1.8 | 0 | 1 | 2 | 3 | 9 |
| Young | CVD | ns | 3.9 | 2.5 | 0 | 2 | 4 | 5 | 17 |
|  |  | cold season | 4.1 | 2.6 | 0 | 2 | 4 | 6 | 17 |
|  |  | warm season | 3.7 | 2.3 | 0 | 2 | 3 | 5 | 14 |
|  |  | low temperature | 4.2 | 2.6 | 0 | 2 | 4 | 6 | 17 |
|  |  | high temperature | 3.7 | 2.3 | 0 | 2 | 3 | 5 | 14 |
|  |  | extreme heat | 3.9 | 2.4 | 1 | 2 | 3 | 5.5 | 11 |
|  | HBP | ns | 0.3 | 0.6 | 0 | 0 | 0 | 1 | 4 |
|  |  | cold season | 0.4 | 0.6 | 0 | 0 | 0 | 1 | 4 |
|  |  | warm season | 0.3 | 0.6 | 0 | 0 | 0 | 1 | 4 |
|  |  | low temperature | 0.4 | 0.6 | 0 | 0 | 0 | 1 | 4 |
|  |  | high temperature | 0.3 | 0.5 | 0 | 0 | 0 | 0 | 3 |
|  |  | extreme heat | 0.3 | 0.6 | 0 | 0 | 0 | 0 | 2 |
|  | IHD | ns | 1.4 | 1.5 | 0 | 0 | 1 | 2 | 10 |
|  |  | cold season | 1.5 | 1.5 | 0 | 0 | 1 | 2 | 10 |
|  |  | warm season | 1.4 | 1.4 | 0 | 0 | 1 | 2 | 7 |
|  |  | low temperature | 1.5 | 1.5 | 0 | 0 | 1 | 2 | 10 |
|  |  | high temperature | 1.4 | 1.4 | 0 | 0 | 1 | 2 | 7 |
|  |  | extreme heat | 1.5 | 1.5 | 0 | 0 | 1 | 3 | 7 |
|  | STR | ns | 1.3 | 1.1 | 0 | 0 | 1 | 2 | 6 |
|  |  | cold season | 1.4 | 1.2 | 0 | 0 | 1 | 2 | 6 |
|  |  | warm season | 1.1 | 1.0 | 0 | 0 | 1 | 2 | 5 |
|  |  | low temperature | 1.4 | 1.2 | 0 | 0 | 1 | 2 | 6 |
|  |  | high temperature | 1.1 | 1.0 | 0 | 0 | 1 | 2 | 6 |
|  |  | extreme heat | 1.3 | 1.0 | 0 | 0.5 | 1 | 2 | 4 |
